# Toll-like receptor activation and gene delivery efficiency in canine dendritic cells: a model for comparative oncology

**DOI:** 10.3389/fimmu.2025.1678896

**Published:** 2025-10-03

**Authors:** Sonia Capellero, Raffaella De Maria, Lisa Adele Piras, Laura Marconato, Lorenza Parisi, Eugenio Mazzone, Caterina Marchiò, Enrico Berrino, Sara Erika Bellomo, Anna Sapino, Giovanni Paolo Stola, Valeria Chiono, Letizia Nicoletti, Luca Aresu

**Affiliations:** ^1^ Department of Veterinary Sciences, University of Turin, Grugliasco, Italy; ^2^ Department of Veterinary Medical Sciences, University of Bologna, Bologna, Italy; ^3^ Department of Medical Sciences, University of Turin, Torino, Italy; ^4^ Candiolo Cancer Institute, FPO-IRCCS, Candiolo, TO, Italy; ^5^ Department of Mechanical and Aerospace Engineering, Politecnico di Torino, Torino, Italy; ^6^ PoliRNA Srl, Torino, Italy

**Keywords:** dendritic cells, tumor-bearing dogs, toll-like receptor, mRNA transfection, lipoplexes

## Abstract

**Objectives:**

Dendritic cells (DCs) are pivotal antigen-presenting cells capable of bridging innate and adaptive immunity, making them promising candidates for cancer immunotherapy. While canine mature DCs (cmDCs) have been successfully generated from circulating mononuclear cells (CMCs) in healthy dogs, their derivation and immunomodulatory capacity in tumor-bearing dogs (TbDs) remain poorly characterized.

**Methods:**

In this study, we evaluated the efficiency of cmDC generation from peripheral blood of both healthy donors (HDs) and TbDs and investigated their functional responses to Toll-like receptor (TLR) agonists and mRNA-based genetic modification.

**Results:**

CD14^+^ monocytes were successfully isolated from peripheral blood using immunomagnetic sorting and differentiated into cmDCs using recombinant canine GM-CSF and IL-4. The differentiation efficiency was comparable between the two groups. In both cohorts, cmDCs upregulated key maturation markers (CD1a, CD80, CD83) and restored DLA class II expression in TbDs. Stimulation with LPS and R848 significantly increased CD80 and CD83 expression and triggered IL-12/p70 and IL-8 production, confirming the acquisition of a functional immunostimulatory phenotype. To assess their amenability to genetic engineering, cmDCs were transfected using DE-DOPE/mRNA lipoplexes. These lipoplexes exhibited favorable physicochemical properties and achieved robust mRNA delivery, resulting in 100% GFP-positive cells and >60% viability, outperforming electroporation in terms of cytocompatibility.

**Conclusions:**

Our findings demonstrate that cmDCs derived from both HDs and TbDs are phenotypically and functionally competent and can be efficiently transfected using a non-viral mRNA delivery system. This strategy offers a viable platform for the development of personalized, DC-based cancer vaccines in canine patients.

## Introduction

Dendritic cells (DCs) are key orchestrators of both innate and adaptive immune responses, functioning as professional antigen-presenting cells (APCs) and serving as vigilant sentinels of the immune system (1–3). Upon exposure to inflammatory stimuli, DCs undergo activation and differentiate into potent APCs capable of initiating robust immune responses (4–6). Within the tumor microenvironment, DCs actively internalize extracellular components, including tumor-associated antigens (TAAs), as well as apoptotic or necrotic tumor cells (7, 8). These antigens are processed intracellularly and presented on major histocompatibility complex (MHC) class I and II molecules, enabling the priming and activation of T lymphocytes and the initiation of antigen-specific anti-tumor immunity (9–11).

Leveraging this immunologic capacity, DCs can be genetically engineered to express defined TAAs or immunomodulatory molecules, to amplify their immunostimulatory potential, and to elicit enhanced anti-tumor responses in cancer patients (12–14). In this context, autologous DCs are often manipulated *ex vivo* and used as cellular vaccines to stimulate specific immune responses (15–17). DCs can be generated from circulating mononuclear cells (CMCs) or bone marrow progenitors, although peripheral blood monocytes remains the preferred source due to its accessibility and lower procedural burden (18). Following *in vitro* differentiation and maturation, DCs can be functionally modified to exhibit heightened immunogenic properties (19–21). Indeed, mature DCs cells upregulate key costimulatory molecules such as CD40, CD80, and CD86, and secrete pro-inflammatory cytokines essential for T-cell activation. These immunostimulatory properties are triggered through the engagement of Toll-like receptors (TLRs) that can be activated by microbial components and complement system, but can also exploited as adjuvants by enhancing the antigen-presenting capabilities and T cell-priming (22).

TLR signaling pathway in DCs cells can be activated *in vitro* through administration of specific immunogenic stimulus, known as TLR agonists. These include lipopolysaccharide (LPS) as TLR4 agonist) and imidazoquinoline compounds such as R848 as TLR7/8 agonist, and polyinosinic:polycytidylic acid (poly I:C) as TLR3 agonist. Activation through these ligands induces distinct cytokine profiles and modulates the expression of MHC molecules and surface markers characteristic of DC maturation, including CD80, CD83, and CD86 (23). As previously described, these immunogenic molecules enhance antigen-specific immune response by facilitating efficient priming of both CD8^+^ cytotoxic and CD4^+^ helper T lymphocytes (9, 24).

In healthy dogs, canine mature dendritic cells (cmDCs) can be successfully generated from CMCs through immunomagnetic enrichment of CD14^+^ monocytes, followed by *in vitro* differentiation using defined cytokine cocktails (25). The cmDCs respond to LPS stimulation by exhibiting morphologic changes and upregulating the transcription of costimulatory molecules (CD80, CD83, CD86) and pro-inflammatory cytokines, indicating effective TLR4 pathway activation (26, 27). However, the generation of cmDCs from tumor-bearing dogs (TbDs) and the immunomodulatory effects of activating multiple TLR pathways such as TLR3, TLR4 and TLR7/8, remain largely unexplored. Investigating these pathways may provide valuable insights into optimizing dendritic cell-based immunotherapies in canine cancers.

This study aimed to assess the *ex vivo* expansion efficiency of cmDCs derived from CMNs isolated from both healthy dogs (HDs) and TbDs. We investigated the biologic effects of TLR agonists, including LPS, R848, and Polyinosinic:polycytidylic acid poly (I:C) administered individually or in combination. The evaluation focused on the production of key immunomodulatory cytokines, such as interleukin-6 (IL-6) and interleukin-12p70 (IL-12p70), as well as phenotypic changes in surface marker expression associated with DC maturation and activation. Lastly, the transfection capability of cmDC was assessed using plasmid vector and mRNA-loaded lipoplexes.

## Materials and methods

### *Ex vivo* isolation and culture of CD14^+^ monocytes from canine peripheral blood samples

Peripheral blood samples (6 mL) were collected in EDTA-coated tubes from both HDs and TbDs following informed owner consent, in accordance with ethical and institutional guidelines of the University of Turin. Peripheral blood mononuclear cells (PBMCs) were isolated immediately using density gradient centrifugation with Lymphoprep™ (Sentinel Diagnostics). Whole blood was gently layered over Lymphoprep and centrifuged at 800×g for 30 minutes at room temperature without brake. The PBMC layer was carefully aspirated and washed twice with phosphate-buffered saline (PBS) at 400×g for 10 minutes to remove residual platelets and plasma components. Cell viability was assessed via trypan blue exclusion, and viable cell numbers were determined using a hemocytometer. CD14^+^ monocytes were then isolated from PBMCs using immunomagnetic separation. Briefly, cells were incubated with anti-canine CD14 microbeads (Miltenyi Biotec) at 4 °C for 30 minutes at a concentration of 20 µL of beads per 10^7^ cells. After incubation, CD14^+^ monocytes were purified using LS columns in a magnetic MACS separator (Miltenyi Biotec), following the manufacturer’s instructions. The purity of the CD14^+^ fraction was verified by flow cytometric analysis. Purified CD14^+^ monocytes were seeded at a density of 2 × 10^6^ cells/mL in 6-well tissue culture plates (Corning) and cultured in Iscove’s Modified Dulbecco’s Medium (IMDM) supplemented with 25 mM HEPES, 2 mM L-glutamine (Euroclone), 100 U/mL penicillin, 100 µg/mL streptomycin (Euroclone), and 10% heat-inactivated fetal bovine serum (FBS; Gibco™).

### Differentiation and maturation of canine monocyte-derived dendritic cells

CD14^+^ monocytes were cultured *in vitro* to induce differentiation into DCs by supplementing the culture medium with recombinant canine granulocyte-macrophage colony-stimulating factor growth factor (GM-CSF, 50 ng/mL) and canine interleukin-4 (IL-4, 10 ng/mL) on day 0 and day 2. On day 5, immature DCs were exposed to a maturation cocktail for 24 hours, consisting of recombinant canine IL-6 (10 ng/mL), canine IL-1β/IL-1F2 (10 ng/mL), canine tumor necrosis factor-α (TNF-α, 15 ng/mL), along with GM-CSF (50 ng/mL) and IL-4 (10 ng/mL) to promote full maturation. All recombinant canine cytokines used for differentiation and maturation were purchased from R&D Systems.

### Multiparameter flow cytometric analysis of dendritic cell phenotype

Immunophenotyping was performed on day 0 (baseline monocytes) and day 6 (mature DCs) to evaluate the expression of surface markers associated with DC differentiation and maturation. A total of 6×10^5^ cells per sample were washed twice with FACS buffer (PBS supplemented with 2% fetal bovine serum and 0.02% sodium azide) and incubated at 4 °C for 30 minutes with a panel of fluorochrome-conjugated monoclonal antibodies targeting CD14, CD40, CD80, CD83, CD86, and CD90. For unconjugated primary antibodies (anti-CD11c, anti-CD1a, and anti-DLA class II), a secondary incubation step was performed using appropriate fluorochrome-conjugated secondary antibodies. Corresponding isotype-matched controls were included for each antibody to assess non-specific binding. Data acquisition was carried out using a BD FACS Celesta flow cytometer (BD Biosciences), calibrated with BD™ CompBeads, with a minimum of 50,000 events recorded per sample. Collected data were analyzed using FlowJo™ software version 10.7.1 (BD Biosciences) to quantify marker expression and evaluate phenotypic changes associated with DC maturation. Mean Fluorescence Intensity (MFI) value was evaluated for statistical analysis.

### Activation of dendritic cells with TLRs agonists

On day 6, following maturation, cmDCs were harvested and reseeded at a density of 1 × 10^6^ cells/mL in 6-well plates. Cells were stimulated with LPS from *Escherichia coli* (10 µg/mL; Merck), the TLR7/8 agonist Resiquimod (R848) (1 µg/mL; Stemcell Technologies), and polyinosinic:polycytidylic acid [poly(I:C)] (50 µg/mL; Merck). During stimulation with TLR antagonists, cells were cultured with IL-6 (10 ng/mL), IL-1β (IL-1F2; 10 ng/mL), TNF-α (15 ng/mL), GM-CSF (50 ng/mL), and IL-4 (10 ng/mL). Stimulation was performed for 24, 48, and 72 hours under standard culture conditions; cmDCs following maturation on day 6 have been used as a normal control. At each time point, cell culture supernatants were collected before and after stimulation and stored at −80 °C for subsequent cytokine quantification. Simultaneously, cells were harvested and processed for flow cytometric analysis to evaluate phenotypic changes induced by TLR engagement. The assay was performed in triplicate from three different HDs.

### Quantitative analysis of cytokine responses of TLR-activated dendritic cells

Cytokine concentrations in cell culture supernatants were quantified using the MILLIPLEX^®^ Canine Cytokine/Chemokine Magnetic Bead Panel-Premixed 13 Plex Space Saver (Merck), which simultaneously detects GM-CSF, IFN-γ, KC-like, IP-10, IL-2, IL-6, IL-7, IL-8, IL-10, IL-15, IL-18, MCP-1, and TNF-α. The assay was performed using the Bio-Plex^®^ 200 System (Bio-Rad), following the manufacturer’s protocol. Data acquisition and analysis were carried out using the Bio-Rad online analysis platform. In addition, IL-12/p70 levels were measured separately using a sandwich enzyme-linked immunosorbent assay (ELISA) specific for canine IL-12/p70 (CUSABIO), according to the manufacturer’s instructions. Absorbance readings were obtained using a microplate reader (Bio-Rad).

### Immunophenotyping of TLR-activated dendritic cells

Immunophenotypic analysis was performed at T0, T24, and T48 hours following stimulation with TLR agonists to assess surface marker modulation on activated DCs. A total of 6×10^5^ cells were washed twice with FACS buffer (PBS supplemented with 2% fetal bovine serum and 0.02% sodium azide) and incubated for 30 minutes at 4 °C with fluorochrome-conjugated monoclonal antibodies targeting CD14, CD80, CD83, and CD86. For unconjugated anti-DLA class II antibodies, a secondary incubation was carried out using appropriate fluorochrome-conjugated secondary antibodies. Corresponding isotype controls were included for each staining condition to evaluate background fluorescence and ensure data specificity. Flow cytometric acquisition was conducted using a BD FACS Celesta™ cytometer, calibrated with BD CompBeads, and a minimum of 50,000 events per sample were recorded. Data were analyzed using FlowJo™ software version 10.7.1 (BD Biosciences) to assess changes in marker expression over time.

### cmDCs transfection by plasmid vector

Mature DCs from six HDs and six TbDs were electroporated by NEON Electroporation System (Invitrogen) following the manufacturer’s protocol using CMV-eGFP kindly gift by J.H (IRCC Candiolo) containing a 741 bp eGFP-encoding fragment whose transcription is controlled by Citomegalovirus (CMV) promoter. Immediately after transfection DCs were resuspended at 5×10^5^ DC/ml in prewarmed (IMDM medium) supplemented with 25 mM HEPES, 2 mM L-glutamine (Euroclone) and 10% heat-inactivated fetal bovine serum (FBS; Gibco™), IL-6 (10 ng/mL), IL-1β/IL-1F2 (10 ng/mL), tumor necrosis factor-α (TNF-α, 15 ng/mL), GM-CSF (50 ng/mL) and IL-4 (10 ng/mL). The viability of DCs was determined by Trypan blue staining and the GFP expression was evaluated with FACS analysis.

### Preparation and physicochemical characterization of messenger RNA loaded lipoplexes

mRNA loaded lipoplexes based on [2-(2,3-didodecyloxypropyl)-hydroxyethyl] ammonium bromide (DE; Nanosoft), and L-alpha-dioleoyl phosphatidylethanolamine (DOPE; Merck), previously developed for microRNA delivery, were formulated. Briefly, empty DE-DOPE liposomes were prepared at a 1:1 w/w ratio using the thin lipid film-hydration method (28–31). The DE-DOPE lipid film was hydrated with Milli-Q water reaching a final concentration of 1 mg∙mL^−1^. Then, DE-DOPE/mRNA lipoplexes were prepared via spontaneous electrostatic interaction between mRNA and the cationic lipid using an amino to phosphate groups (N/P) ratio of 6. DE-DOPE/mRNA lipoplexes were prepared at two different mRNA concentrations: 1X corresponding to 0.5 μg mRNA and 9.3 μg DE-DOPE, and 2X corresponding to 1 μg mRNA and 18.6 μg DE-DOPE. Cyanine-5 labelled mRNA (Cy5-mRNA, OZBiosciences) and mRNA expressing green fluorescent protein (GFP-mRNA, OZBiosciences) were loaded into DE-DOPE lipoplexes. The lipoplexes were characterized for their hydrodynamic size, Z-potential and polydispersity index (PDI) by Dynamic Light Scattering (DLS; Litesizer, Anton Paar). Loading efficiency (LE) was characterized by quantifying the amount of unloaded Cy5-mRNA after complexation through UV-Vis spectroscopy.


$$LE\;\left(\%\right)=\frac{Total\;mRNA-free\;mRNA\;in\;the\;surnatant}{Total\;mRNA}\;$$

### cmDCs transfection by mRNA loaded lipoplexes

Mature DCs from six HDs were transfected with Cy5 and GFP mRNA loaded lipoplexes. A total of 100.000 mature DCs/well were seeded on IBIDI μ-slide 8 well and transfected with DE-DOPE/Cy5-mRNA lipoplexes and DE-DOPE/GFP-mRNA lipoplexes at 1X and 2X concentrations. After incubation for 5 hours, they were fixed with PAF 4% (paraformaldehyde solution) for 15 minutes at 4 °C and stained with SPY620-DNA for nuclei (blue) and WheatGerm Agglutinin (green) AlexaFluor488 for membrane (WGA only in Cy5-mRNA lipoplexes). Immunofluorescence images were acquired on TCS SP8 confocal laser‐scanning microscopes (LAS AF software, Leica Microsystems). Different fields per sample were randomly chosen for analysis.

### Statistical analysis

Normality of the data for each group was assessed using the Shapiro-Wilk test. To evaluate the homogeneity of variances, an F-test was conducted. When both assumptions—normal distribution and equal variances—were satisfied, Student’s t-tests were used to compare group means. In cases where data deviated from normality or showed unequal variances, the Wilcoxon rank-sum test was employed as a non-parametric alternative. All resulting p-values were subjected to *post-hoc* correction using the False Discovery Rate (FDR) method, and results were considered statistically significant if the adjusted p-value (q-value) was ≤ 0.05. For the ELISA analyses, expression levels (percentages) of surface markers and cytokine concentrations (pg/mL) were integrated across different time points or experimental conditions (e.g., healthy vs. cancer). ANOVA was used to assess differences across analytes, while t-tests were used to compare MFI values between specific groups. All statistical analyses were performed by R software (v4.3.3).

## Results

### Canine patients selection

Peripheral blood samples were collected from a total of 18 dogs, including 9 TbDs and 9 HDs. In all cases, mature DCs were successfully generated from PBMCs using an established *ex vivo* differentiation protocol. The tumor-bearing cohort encompassed a range of neoplastic conditions, including three cases of T-cell lymphoma, two cases of diffuse large B-cell lymphoma, and one case each of fibrosarcoma, oral melanoma, cutaneous mast cell tumor, and splenic hemangiosarcoma.

### Generation and characterization of CD14^+^ CMCs as precursor of mature dendritic cells

**Figure 1:**
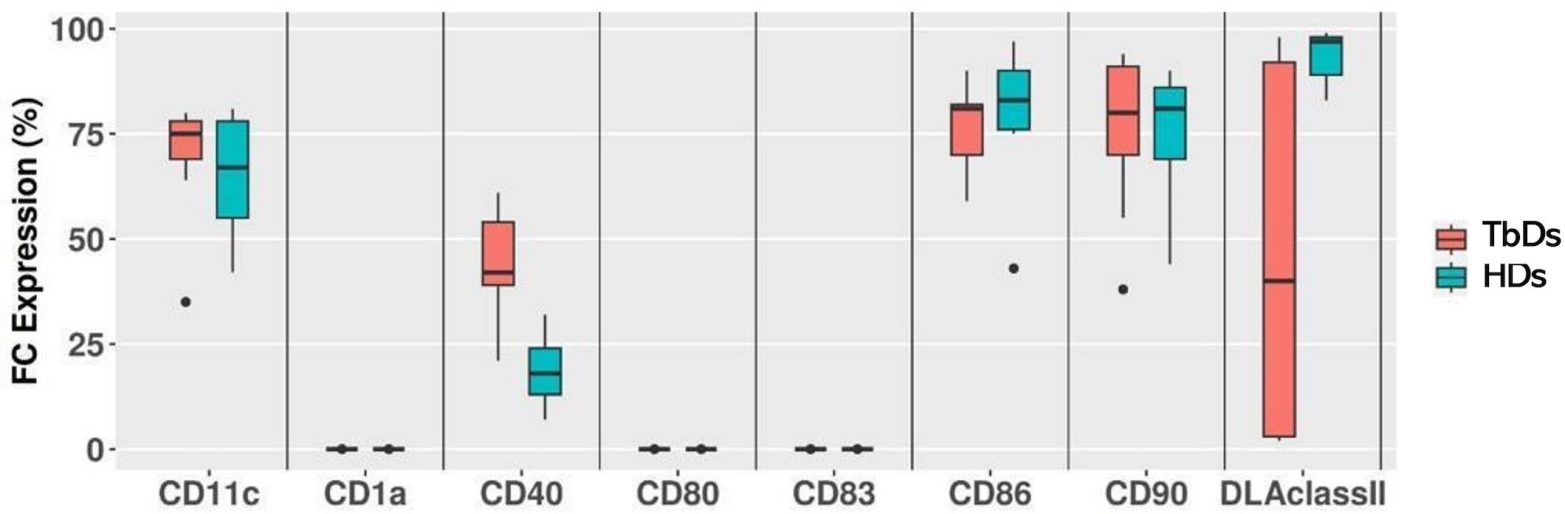
Flow cytometry immunophenotyping of CD14+ monocytes at day 0. Median expression levels of monocyte marker CD14^+^ and dendritic markers CD1a, C11c, CD40, CD80, CD83, CD86, CD90 and DLA class II in circulating monocytes after immunomagnetic separation from healthy dogs (HD) and tumor bearing dogs (TbD) at time day 0 (T0).

The immunomagnetic separation using CD14-specific microbeads demonstrated efficient isolation of monocytes from PBMCs. At baseline (T0), CD14^+^ monocytes accounted for a mean of 11.1% of the total PBMC population, with no significant differences (p>0.05) between HDs (10.7%) and TbDs (11.5%). Following the 6-day differentiation and maturation protocol, the resulting DC population constituted approximately 74% of the cultured cells. Differentiation efficiency was comparable between groups, with cultures derived from HDs and TbD generating 74.2% and 73.9% phenotypically mature DCs, respectively. Detailed cell counts for each phase of isolation and differentiation across all individual donors are provided in **Supplementary Table 1**. Immunophenotypic analysis at T0 revealed a significant difference between the two groups in the expression of CD40, a critical costimulatory molecule involved in dendritic cell activation and T-cell priming. CD14^+^ monocytes isolated from TbDs exhibited reduced surface expression of CD40 (median: 18%, range: 7–32%) compared to those from HDs (median: 42%, range: 21–61%) (p < 0.005). In contrast, CD86, CD11c, and CD90 were consistently expressed with no significant differences, while CD1a, CD80, and CD83 were uniformly undetectable in both groups (**Figure 1**).

### Phenotypic modification of dendritic cells after differentiation

**Figure 2:**
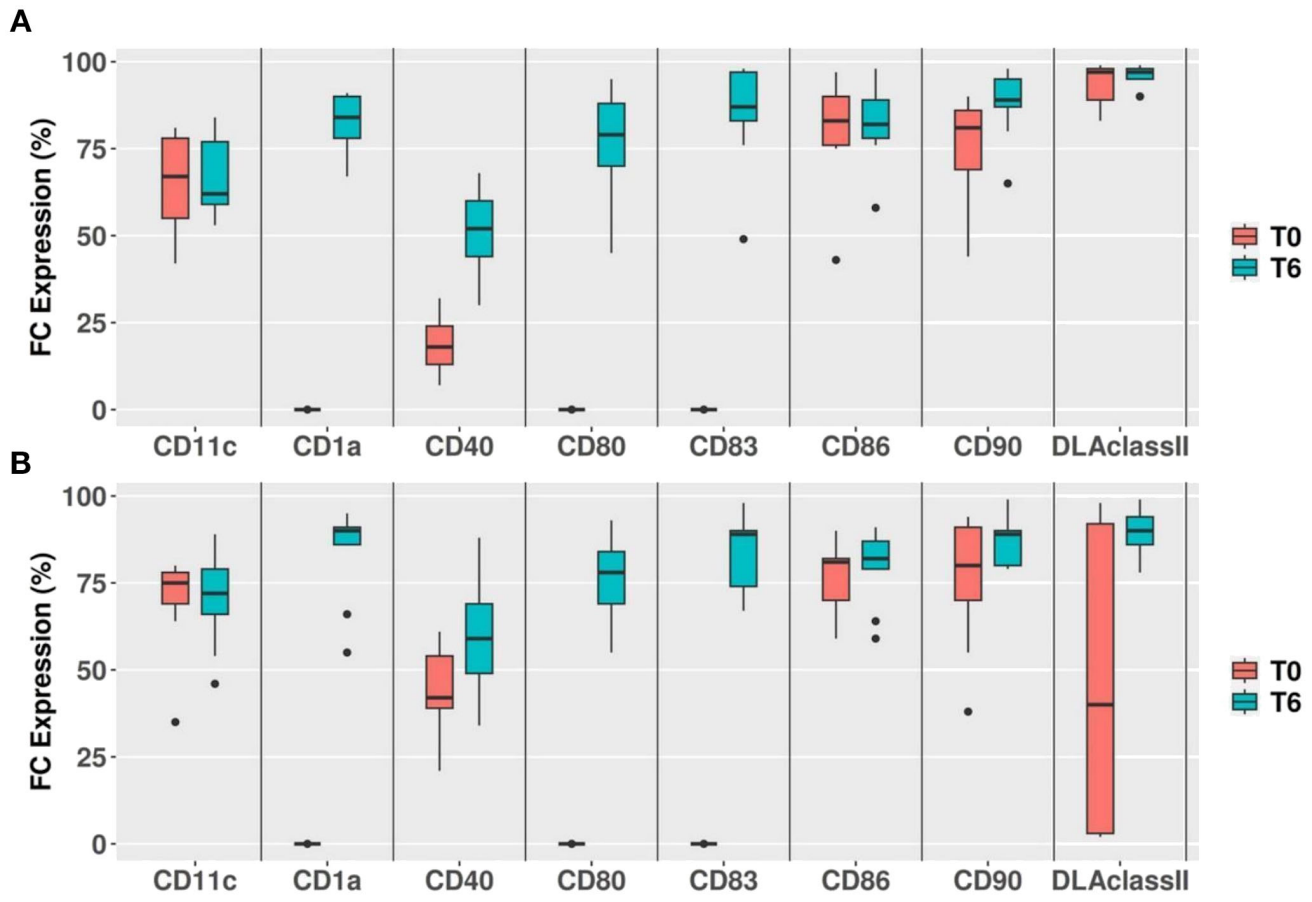
Flow cytometry immunophenotyping of mature dendritic cells at day 6. Flow cytometry immunophenotyping of mature dendritic cells at day 6. Fold changes in mean fluorescence intensity (MFI) of CD1a, CD11c, CD40, CD80, CD83, CD86, CD90, and DLA class II in mature dendritic cells from healthy donors (HDs, 2A) and tumour bearing dogs (TbDs, 2B) after 6 days of *in vitro* differentiation (T6). Significant upregulation of CD1a, CD80, and CD83 was observed in both HDs and TbDs samples compared to baseline (p< 0.002).

After 6 days of culture, cmDCs were successfully generated from both HDs and TbDs groups. Differentiated cells exhibited a significant upregulation of CD1a, CD80, and CD83 compared to baseline monocyte levels (p < 0.002), consistent with the acquisition of a mature dendritic cell phenotype. Importantly, DLA class II expression, which was markedly diminished at baseline in four TbD cases, was fully restored following differentiation, reaching levels comparable to those observed in the remaining TbDs. This recovery suggests that *in vitro* cytokine-driven differentiation is capable of reversing tumor-associated suppression of MHC class II expression in monocyte precursors. Results are represented in **Figures 2A, B**.

### Phenotypic changes of dendritic cells following TLR agonists activation

Stimulation of cmDCs with TLR agonists induced distinct phenotypic changes. Flow cytometry analysis showed a statistically significant increase in the mean fluorescence intensity (MFI) of CD80 and CD83 following stimulation with R848 and LPS at all time points examined (ANOVA test, *p* < 0.05). In contrast, CD86 and DLA class II expression remained unmodified throughout the stimulation period. Conversely, treatment with poly(I:C) did not alter the phenotypic profile of cmDCs under any experimental condition. These results are shown in **Table 1** and **Supplementary Figure 1**.

**Table 1:** Phenotypic changes of dendritic cells following TLR agonists.

Markers	Condition	F value	Pr (>F)
CD 80	LPS	6.24	**0.02**
CD83	R848	5.37	**0.03**
CD83	LPS	3.23	0.08
CD 80	POLY	2.15	0.17
CD83	POLY	1.95	0.20
DLA class II	R848	1.70	0.24
CD 80	R848	1.63	0.26
CD86	R848	1.56	0.27
DLA class II	POLY	1.08	0.41
CD86	POLY	0.70	0.58
DLA class II	LPS	0.13	0.94
CD86	LPS	0.06	0.98

Membrane markers with changes in mean fluorescence intensity (MFI) in response to LPS, R848, or Poly(I:C). CD80 and CD83 show a significant increase in MFI following R848 and LPS stimulation (ANOVA, *p* < 0.05), while Poly(I:C) (bold characters).

### Cytokine profiles following dendritic cells activation by TLR agonists

**Figure 3:**
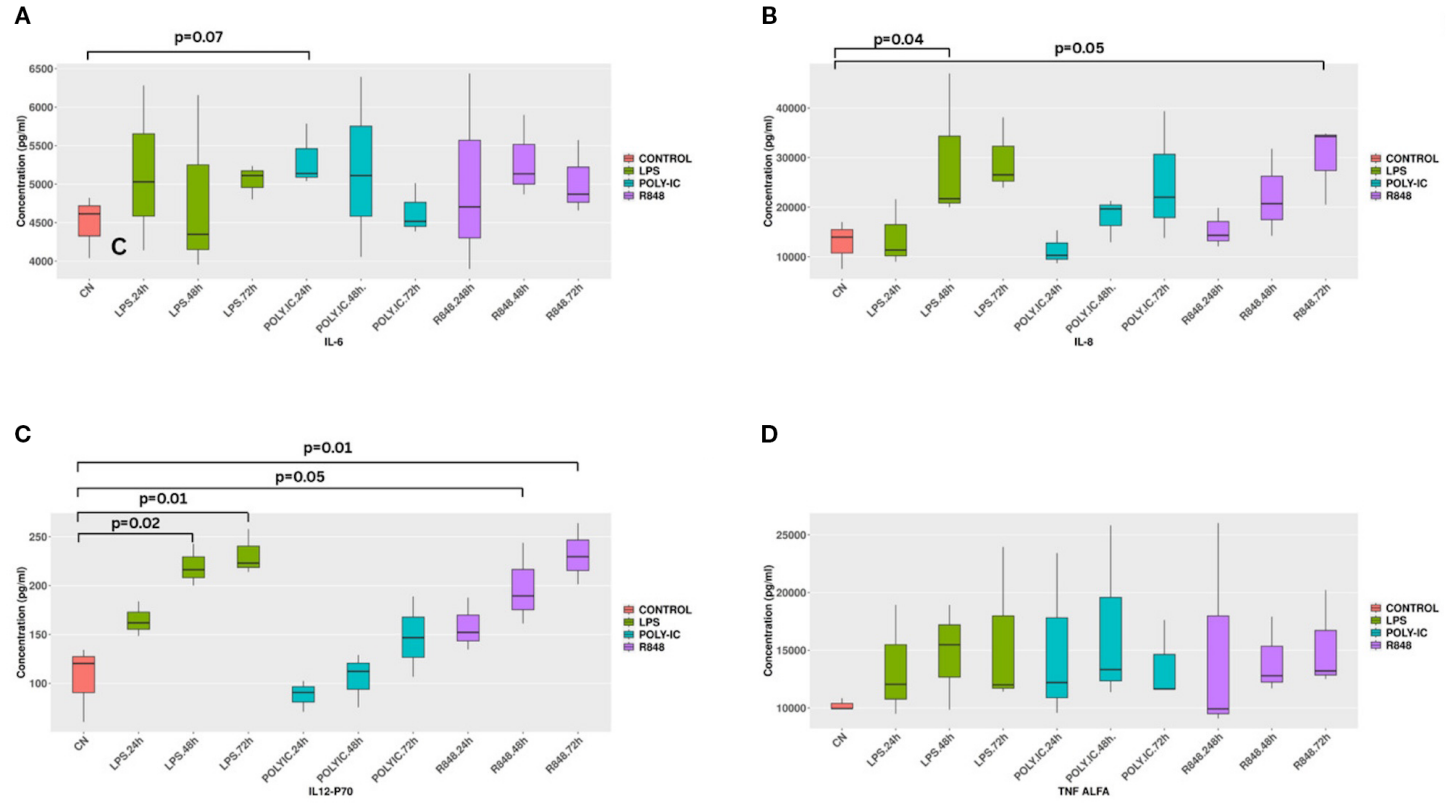
Cytokine Production after *in vitro* stimulation. Cytokines production after stimulations with LPS and R848. LPS stimulation for 48 hours and R848 stimulation for 72 hours induce a significant increase of IL8 (*p* < 0.04 and p< 0.05) (3B). R848 and LPS stimulation for 48 hours significantly increases IL-12p70 levels (p< 0.05 and p< 0.02) respectively. After 72 hours a significant higher increase of IL-12p70 was found (p< 0.02) for both the molecules (3C). IL-6 and TNF alpha do not show any significant change in all considered conditions.

Stimulation with LPS for 48 hours and resiquimod (R848) for 72 hours resulted in a significant increase in IL-8 concentrations compared to unstimulated controls (*p* < 0.05) (**Figure 3B**). Although IL-6 and TNF-α levels consistently increased following stimulation with both agonists, a statistical significance was not reached (**Figures 3A, D**). Additional cytokines, including GM-CSF, IFN-γ, KC-like, IP-10, IL-2, IL-7, IL-10, IL-15, IL-18, and MCP-1, were detected at low concentrations and their expression remained unaltered after stimulation (data not shown). To assess whether stimulation with distinct immunogens at different time points modulates IL-12p70 production, responses following exposure to R848, LPS, and poly I:C were evaluated. Stimulation with R848 and LPS at 48 hours resulted in a significant increase in IL-12p70 levels (*p* < 0.05 and *p* < 0.02, respectively). At 72 hours, both immunogens continued to elicit a significant elevation in IL-12p70 production (*p* < 0.02), as shown in **Figure 3C**. In contrast, stimulation with poly I:C did not induce a significant change in IL-12p70 levels at either time point.

### Physicochemical properties of mRNA loaded lipoplexes

mRNA loaded lipoplexes displayed a hydrodynamic diameter of 343 ± 15 nm, PDI of 0.17 ± 0.06 and a zeta potential of 37 ± 2 mV, similarly to those of miRNA loaded lipoplexes previously developed^30^. LE of mRNA in DE-DOPE lipoplexes, evaluated by UV-Vis spectroscopy, was 99%, as for miRNA delivery.

### Enhanced viability and transgene expression in cmDCs via lipoplexes delivery

**Figure 4:**
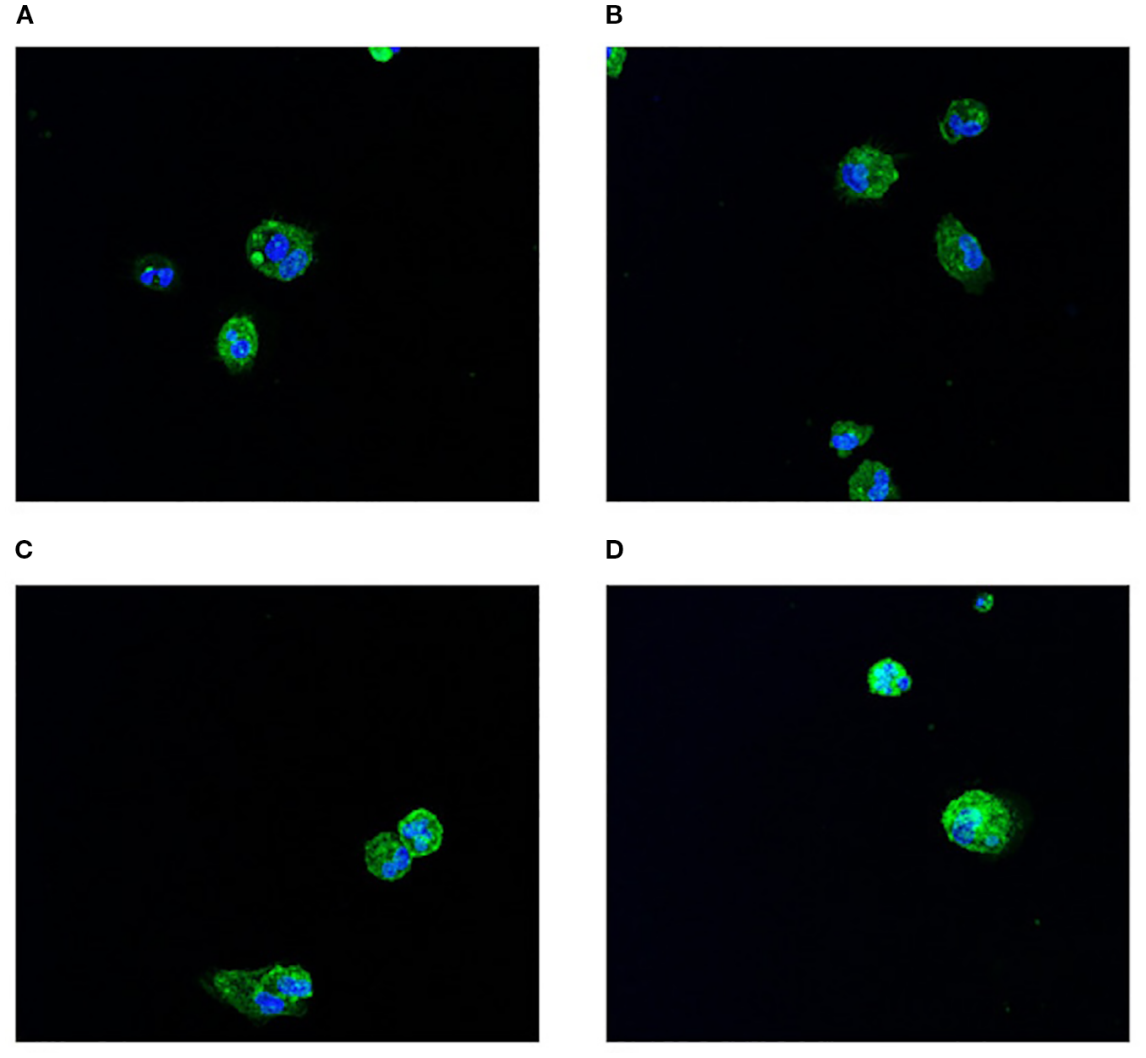
Immunofluorescence analysis of cmDCs following DE-DOPE lipoplex transfection. Panels **(A, B)** display representative images of cmDCs (green fluorescence-positive) transfected with 1X DE-DOPE lipoplexes after 5 hours. Panels **(C, D)** show representative images of cmDCs transfected with 2X DE-DOPE lipoplexes after 5 hours. Nuclei were counterstained with SPY620-DNA. Images were acquired at 63× magnification.

**Figure 5:**
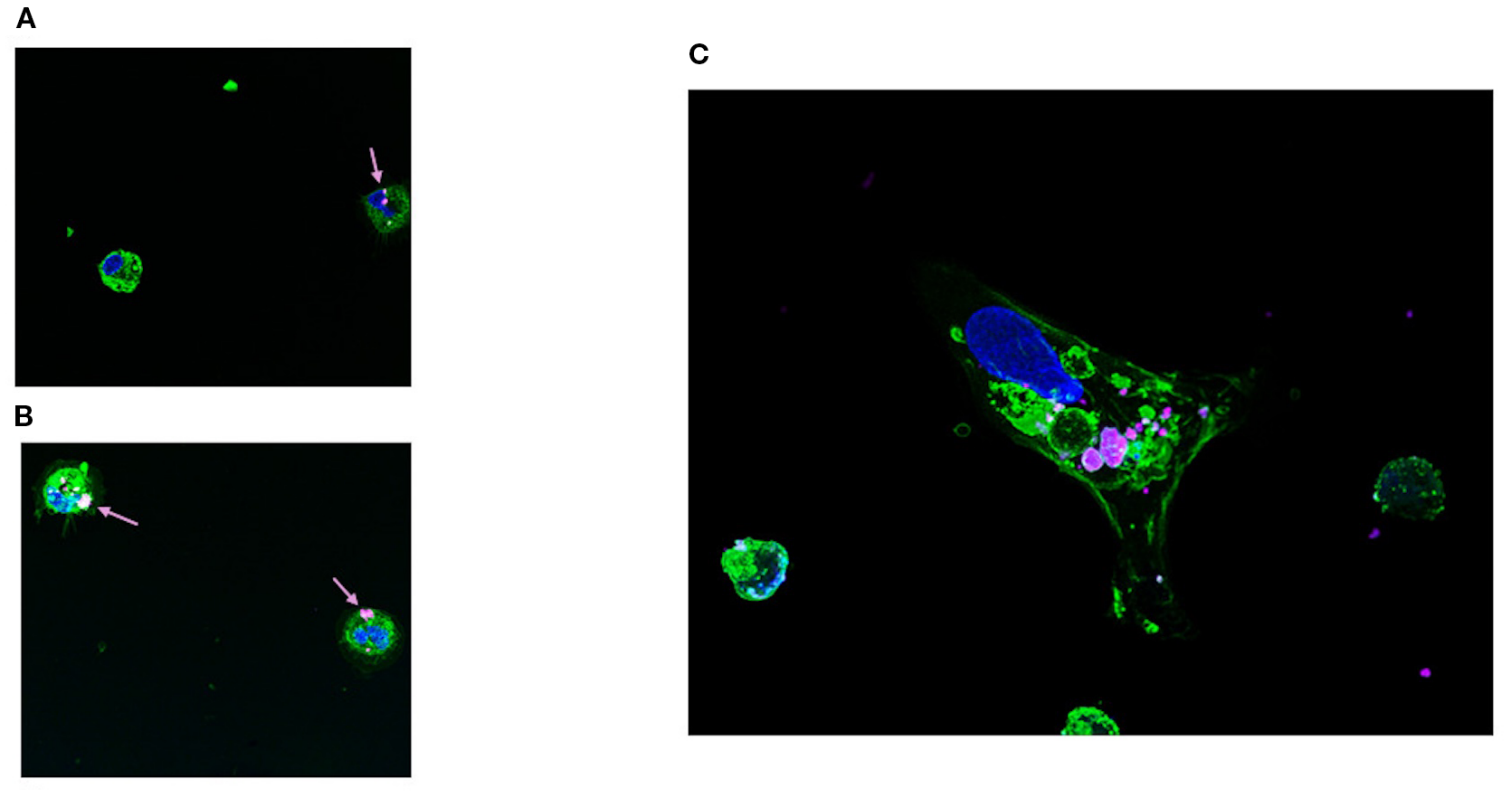
Immunofluorescence analysis of Cy5-labeled mRNA delivered via DE-DOPE lipoplexes. Panels **(A, B)** show representative fluorescence microscopy images of cmDCs transfected with Cy5-labeled mRNA (magenta) delivered by 1X and 2X DE-DOPE lipoplexes (arrows), respectively, after 5 hours. Panel **(C)** provides a magnified view highlighting Cy5-mRNA localization. Nuclei were stained with SPY620-DNA (blue) and cell membranes with Wheat Germ Agglutinin-AlexaFluor488 (green). Images were acquired at 63× magnification..

Electroporation of cmDCs with a GFP-expressing vector resulted in an average post-transfection mortality exceeding 70%, with GFP expression detected at low level in the remaining viable cells (data not shown). In contrast, cmDCs transfected using DE-DOPE/Cy5 mRNA lipoplexes exhibited a higher average cell viability of 52% and 62% after 5h transfection with 1X and 2X lipoplexes concentrations, respectively. The formulated lipoplexes displayed a hydrodynamic diameter of 343 ± 15 nm, PDI of 0.17 ± 0.06 and a zeta potential of 37 ± 2 mV, with a LE% of approximately 99%. **Figure 4** shows the efficiency of GFP expression at 1X nanoparticle concentrations (**Figures 4A, B**) and 2X nanoparticle concentrations (**Figures 4 C, D**). Notably, fluorescence intensity was higher in cmDCs treated with DE-DOPE/GFP-mRNA lipoplexes at 2X concentration, indicating more efficient transgene expression. Confocal microscopy analyses confirmed that Cy5-mRNA loaded lipoplexes were successfully internalized by cmDCs via phagocytosis (**Figures 5 A–C**).

## Discussion

Dendritic cells represent professional antigen-presenting cells that serve as central orchestrators of adaptive immunity, bridging innate immune surveillance with antigen-specific T-cell responses through their unique capacity for cross-presentation and co-stimulation (32–34). This distinctive immunologic condition has established DCs as compelling therapeutic agents in cancer immunotherapy protocols (35, 36). Due to their specialized cellular machinery, DCs are considered ideal candidates for adoptive cell therapy and the development of personalized cancer vaccines (2, 37). Of particular significance is their capacity to process cancer-specific neoantigens, enabling highly selective targeting of malignant tissue (38, 39). While cmDCs have previously been generated from CMCs in HDs, the efficiency of cmDCs generation in TbDs, as well as their functional responsiveness to TLR, remains largely uncharacterized. In this study, we achieved comparable efficiency in the isolation and differentiation of monocytes into DCs from PBMCs obtained from HDs and TbDs. Monocyte recovery and subsequent differentiation into DCs were highly consistent in both groups, irrespective of disease status or cancer histotype, with a mean differentiation efficiency of 74% following a 6-day culture period. Interestingly, in contrast to previously described protocols (26, 27), full *ex vivo* maturation was successfully attained by supplementing the culture system with recombinant canine GM-CSF. Following immunomagnetic sorting, CMCs expressed characteristic monocyte markers, including CD11c, CD86, and CD90 and after six days of differentiation a marked upregulation of CD1a, CD80, and CD83 was shown. Notably, these markers were further upregulated *in vitro* using LPS and R848. Indeed, both CD80 and CD83 expression levels were significantly upregulated, confirming cellular response to microbial components and underlining the activation potential of canine DCs. These findings suggest that LPS and R848 may serve as effective adjuvants by enhancing the antigen-presenting capacity and T cell-priming functionality of cmDCs.

Quantitative cytokine analysis revealed also that LPS and R848 stimulation induced a significant increase in IL-8 secretion, one of the key pro-inflammatory cytokines responsible for neutrophil recruitment to sites of inflammation (40, 41).Although the increase in IL-6 production over time did not reach significance, a clear time-dependent trend was observed. This result is consistent with expectations (42), as IL-6 is already present in the cmDCs differentiation medium, likely masking measurable differences. Furthermore, stimulation with LPS and R848 resulted in a significant upregulation of IL-12/p70. This cytokine plays a central role in cell-mediated immunity by promoting Th1 differentiation, activating macrophages and NK cells, and its increased production confirms the generation of functional DCs in the culture system (24). Based on these preliminary findings, we hypothesize that TLR engagement during dendritic-cell maturation upregulates MHC I/II and co-stimulatory molecules (CD80/CD86, CD40), boosts secretion of Th1-polarising cytokines (IL-12p70, type I IFNs), enhances cross-presentation of tumour antigens, and promotes CCR7-dependent lymph-node homing while reducing susceptibility to tumour-derived tolerogenic cues, thereby improving priming of cytotoxic T cells and activation of NK cells against cancer.

A major challenge in T-cell-based immunotherapy lies in the efficient priming of antigen-specific T-cell responses by DCs presenting defined peptide or mRNA-encoded antigens, including cancer neoantigens (39, 43). Lipid lipoplexes have emerged here as effective non-viral mRNA delivery platforms due to their ability to protect mRNA, enhance uptake, and promote endosomal escape (26, 27). We evaluated DE-DOPE/miRNA lipoplexes, previously validated for efficient, biocompatible miRNA delivery in cardiac fibroblasts, as a delivery system for mRNA transfection in DCs. DE-DOPE/mRNA lipoplexes (N/P=6) retained favorable physicochemical properties (hydrodynamic diameter ~343 nm, PDI 0.167, zeta potential +37 mV, LE% ~99%) and demonstrated high transfection efficiency in canine DCs. The DCs internalized Cy5-labeled mRNA lipoplexes effectively, and DE-DOPE/GFP-mRNA delivery resulted in 100% GFP-positive cells while maintaining >60% cell viability. Although cytocompatibility was slightly below the ISO 10993–5 threshold of 70%, optimization of the mRNA dose or N/P ratio in future may further improve tolerability without compromising transfection efficiency. These preliminary findings highlight the potential of DE-DOPE lipoplexes as efficient, cytocompatible carriers for mRNA-based DC engineering, offering a promising strategy for improving DC-based vaccines in canine cancers or other diseases.

In conclusion, we have shown comparable efficiency in generating cmDCs from both healthy and tumor-bearing dogs. Using recombinant canine cytokines, we successfully induced the differentiation of cmDCs displaying mature phenotypes, characterized by robust expression of antigen-presenting and co-stimulatory molecules that are known features consistent with those observed in human DCs. Moreover, *in vitro* stimulation with TLR agonists further enhanced the expression of key co-stimulatory markers, thereby improving the capacity of DCs to activate T-cell responses. Despite few limitations in our study, including the limited number of cancer histotypes analyzed, the reduced number of experiments conducted with DE-DOPE/mRNA lipoplexes, and the absence of functional assays demonstrating effective T-cell activation, our findings underscore the potential of cmDCs as a viable platform for the development of T cell-based immunotherapies in the canine model. Moreover, the successful delivery of mRNA using DE-DOPE lipoplexes represents a promising approach for the genetic modification of cmDCs, paving the way for refined and personalized DC-based cancer immunotherapy strategies.

## Data Availability

The raw data supporting the conclusions of this article will be made available by the authors, without undue reservation.

## Supplementary material

Supplementary Figure 1 Mean Fluorescence Values (MFI) of CD80 and CD83, CD86 and DLA Class II following stimulation with R848 and LPS at all time points examined (ANOVA test, p< 0.05).
