# Supplementary figures and images for: Toll-like receptor activation and gene delivery efficiency in canine dendritic cells: a model for comparative oncology
Source: Front Immunol. 2025 Oct 3;16:1678896. doi: 10.3389/fimmu.2025.1678896 (PMC12531200; doi:10.3389/fimmu.2025.1678896)

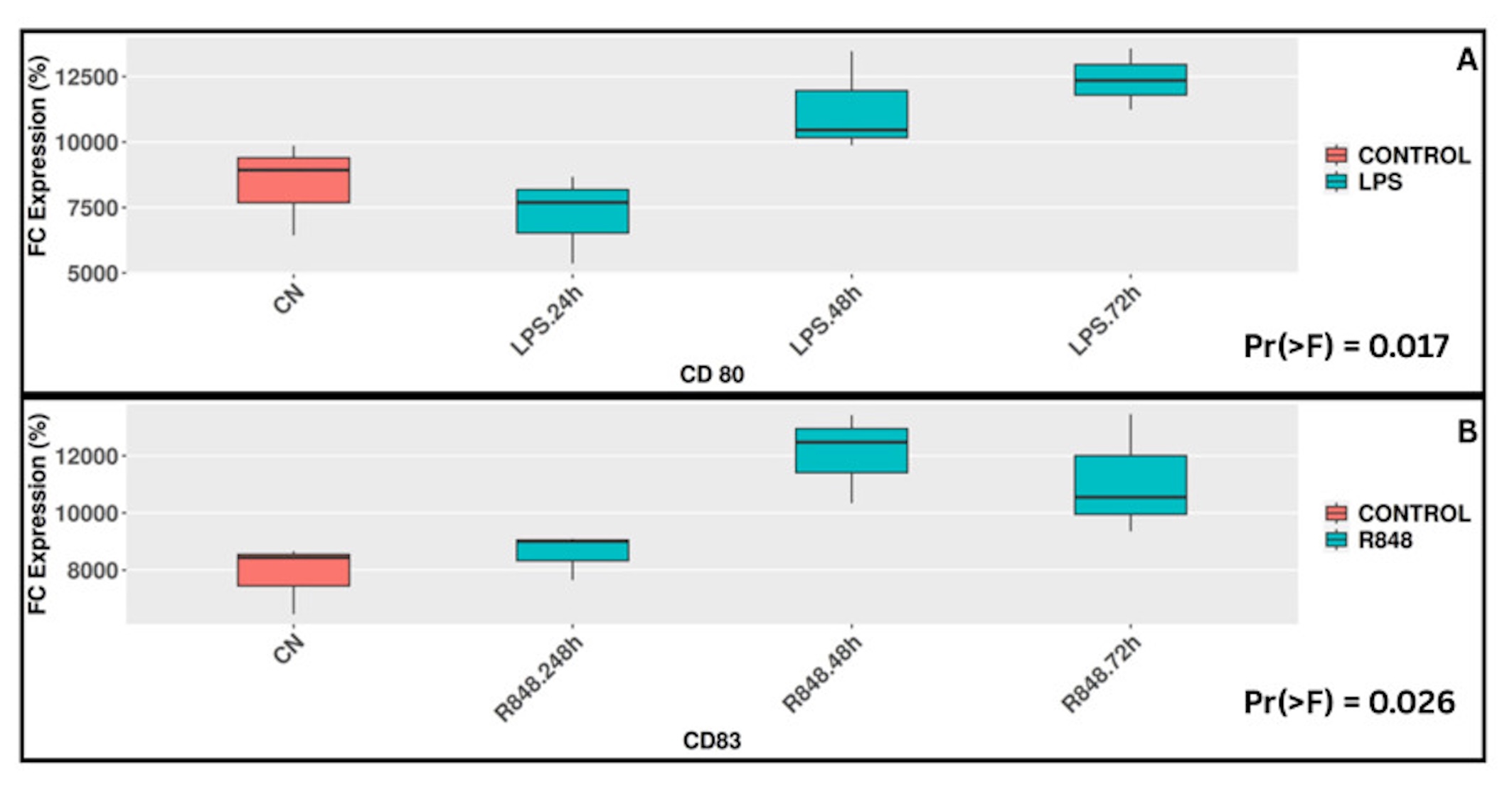

Supplement: Supplementary Figure 1 — Mean Fluorescence Values (MFI) of CD80 and CD83, CD86 and DLA Class II following stimulation with R848 and LPS at all time points examined (ANOVA test, p< 0.05). [file Image1.jpeg]
