# Supplementary material for: Toll-like receptor activation and gene delivery efficiency in canine dendritic cells: a model for comparative oncology
Source: Front Immunol. 2025 Oct 3;16:1678896. doi: 10.3389/fimmu.2025.1678896 (PMC12531200; doi:10.3389/fimmu.2025.1678896)
Supplement: Supplementary file 2 [file Table1.docx]

**Supplementary Table 1**

|  | **T0** | | | **T6** | |
| --- | --- | --- | --- | --- | --- |
| **Donors** | **PBMC** | **CD14+** | **% CD14** | **mature DC** | **% mature DC** |
| **HD1** | 25x10^6 | 2.8x10^6 | 11.2 | 2.4x10^6 | 85.7 |
| **HD2** | 15x10^6 | 3x10^6 | 20.0 | 2.7x10^6 | 91.3 |
| **HD3** | 21x10^6 | 1.9x10^6 | 9.0 | 1.3x10^6 | 68.4 |
| **HD4** | 9x10^6 | 7.5x10^5 | 8.3 | 4.2x10^5 | 56.0 |
| **HD5** | 12x10^6 | 9x10^5 | 7.5 | 8.4x10^5 | 93.7 |
| **HD6** | 16x10^6 | 2x10^6 | 12.5 | 1.4x10^6 | 71.0 |
| **HD7** | 7x10^6 | 5.6x10^5 | 8.1 | 4.8x10^5 | 86.2 |
| **HD8** | 8x10^6 | 7.9x10^5 | 9.9 | 3.2x10^5 | 40.3 |
| **HD9** | 16x10^6 | 1.5 x10^6 | 9.4 | 1.3x10^6 | 75.3 |
| **TbD 1** | 15x10^6 | 2.3 x10^6 | 15.3 | 1.8x10^6 | 82.2 |
| **TbD 2** | 11x10^6 | 1.3x10^6 | 11.8 | 8.6x10^5 | 66.2 |
| **TbD 3** | 14x10^6 | 1.4x10^6 | 10.0 | 9.8x10^5 | 70.6 |
| **TbD 4** | 28x10^6 | 2x10^6 | 7.1 | 1.05x10^6 | 52.5 |
| **TbD 5** | 12x10^6 | 7.5x10^5 | 6.3 | 5.1x10^5 | 68.7 |
| **TbD 6** | 14x10^6 | 8,9 x10^5 | 6.4 | 7.3x10^5 | 82.6 |
| **TbD 7** | 20x10^6 | 5x10^6 | 25.0 | 4.3x10^6 | 87.2 |
| **TbD 8** | 22 x10^6 | 2.5x10^6 | 11.4 | 2.1x10^6 | 84.0 |
| **TbD 9** | 15 x10^6 | 1.6x10^6 | 10.7 | 1.3x10^6 | 70.9 |
